# Supplementary material for: HIV-1 targets L-selectin for adhesion and induces its shedding for viral release
Source: Nat Commun. 2018 Jul 19;9:2825. doi: 10.1038/s41467-018-05197-2 (PMC6053365; doi:10.1038/s41467-018-05197-2)
Supplement: Supplementary file 1 — Supplementary Information [file 41467_2018_5197_MOESM1_ESM.pdf]

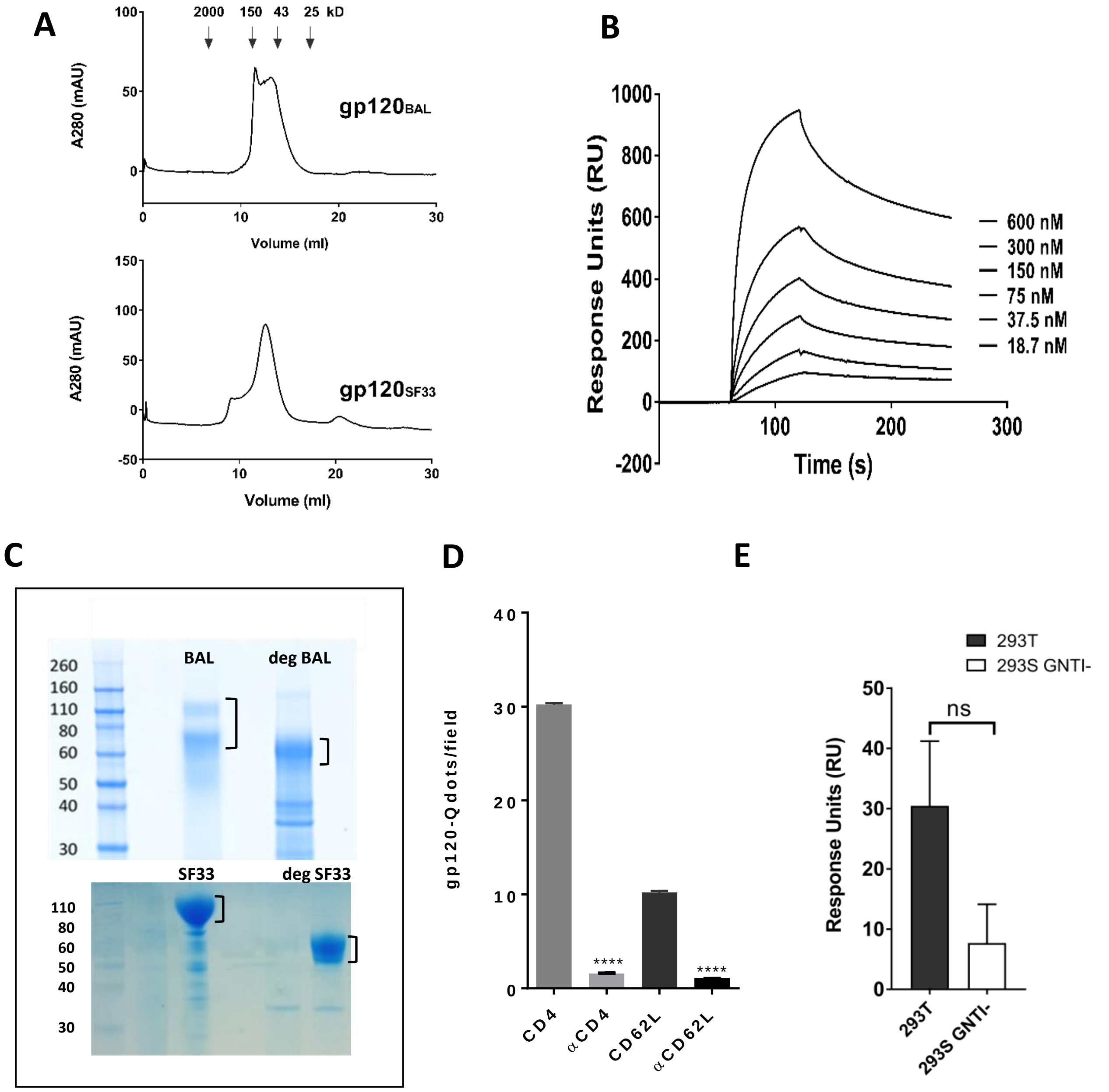

Supplementary Figure 1. Binding of recombinant gp120 to CD62L. (A) Size exclusion chromatograms of 293T cell expressed recombinant gp120 protein from R5-tropic BAL (top) and X4-tropic SF33 (bottom) strains. The retention volumes for molecular weight standards are indicated on the top chromatogram. (B) BIAcore sensorgrams show the binding of recombinant gp120 from X4 tropic strain SF33 to immobilized CD62L with a calculated dissociation constant of ~100 nM. (C) Deglycosylation of recombinant $gp120_{BAL}$ (upper panel) and $gp120_{SF33}$ (lower panel) by PNGase. Lanes are indicated from left to right for molecular weight marker, native and PNGase treated gp120. (D) Binding of fluorescence labeled gp120-Qdots to plate-bound CD4 or CD62L in the absence and presence of their blocking antibodies. (E) BIAcore response of 0.6uM recombinant $gp120_{SF33}$ expressed from either 293T (black) or

293S GnTI$^-$ (open) cells binding to immobilized CD62L-Fc. The CD62L binding affinity for 293S GnTI$^-$ expressed gp120$_{SF33}$ is ~350 nM. All panels in this figure are from at least two independent experiments.

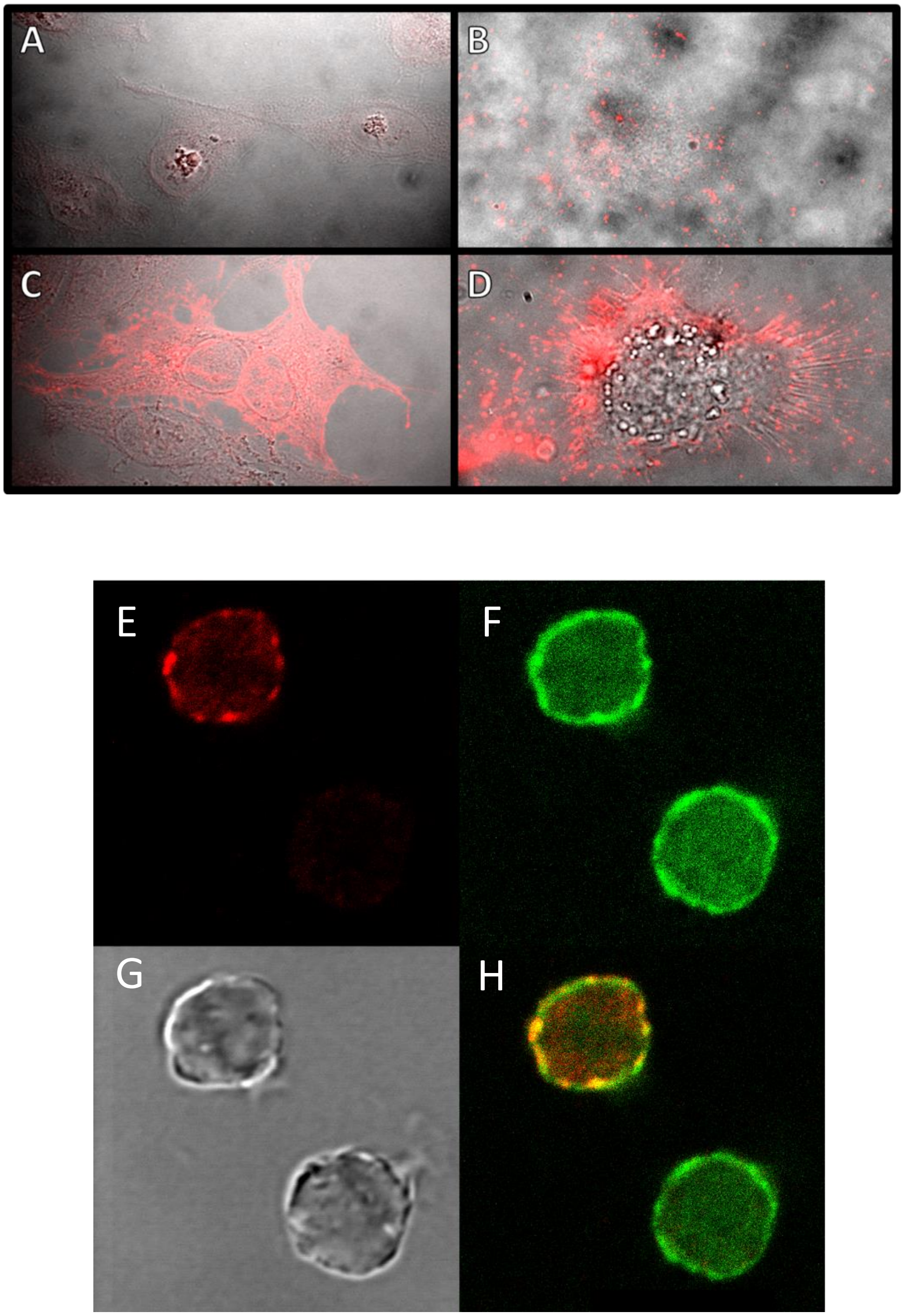

Supplementary Figure 2. Cell surface L-selectin expression and gp120 binding. Fluorescence microscope images of untransfected (A,B) and CD62L-transfected (C,D) Hela cells stained with anti-CD62L antibody (A,C) or gp120-Qdots (B,D). E-H) A montage of primary CD4$^+$ T cells stained for CD62L (E, red), CD4 (F, green), DIC (G, gray), and a merge of the CD4 and CD62L channels (H). All images are representative of at least three images in each category.

A

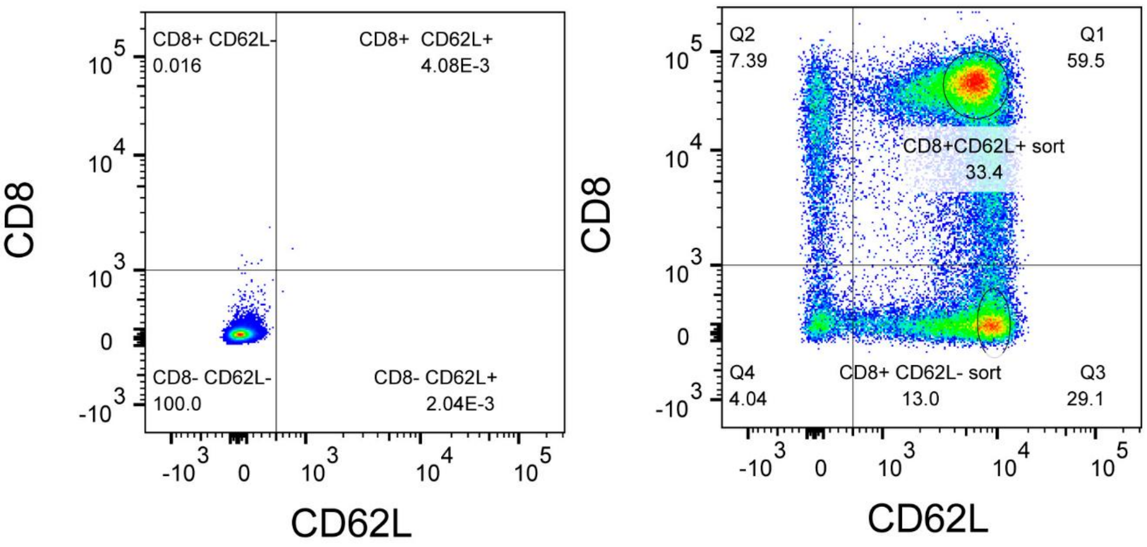

Unstained Control

Stained Sample for Sort

B

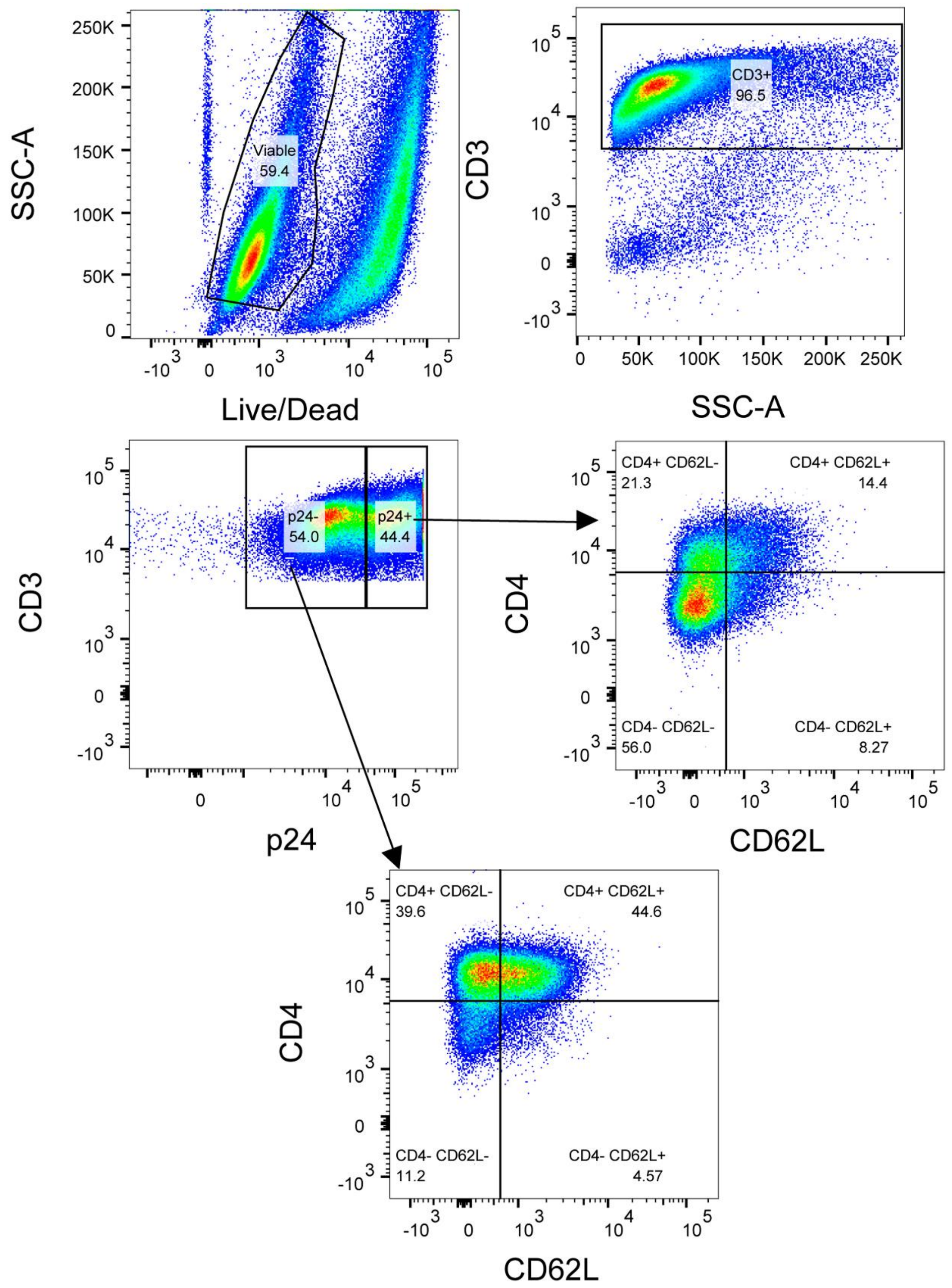

Supplementary Figure 3. Flow cytometry gating schemes. A) Sorting of CD62L$^+$ and CD62L$^-$ CD8$^+$ T cells for HIV-1$_{BAL}$ pulldown assay. CD8$^+$ T cells were enriched from PBMCs using the Stemcell EasySep™ Human CD8+ T Cell Enrichment Kit, and then stained with CD8 and CD62L antibodies (BD Bioscience) prior to sorting on CD62L$^+$CD8$^+$or CD62L$^-$CD8$^+$cells. The sorted cells were used in Figure 1H. The results are representative of two independent experiments. B) Gating scheme used in analysis of HIV-1

infection. The infected or uninfected cells were stained with Live/Dead compound, CD3, CD4, CD62L, and p24. The samples were acquired on a BD Canto and analyzed using FlowJo software. Samples were gated on viability based on the Live/Dead compound. Then the viable cells were gated on CD3, followed by p24+ or p24- staining within the CD3 population. In each of the p24+ or p24- populations cells were examined for CD4 and CD62L expression. Gates were set using isotype controls.

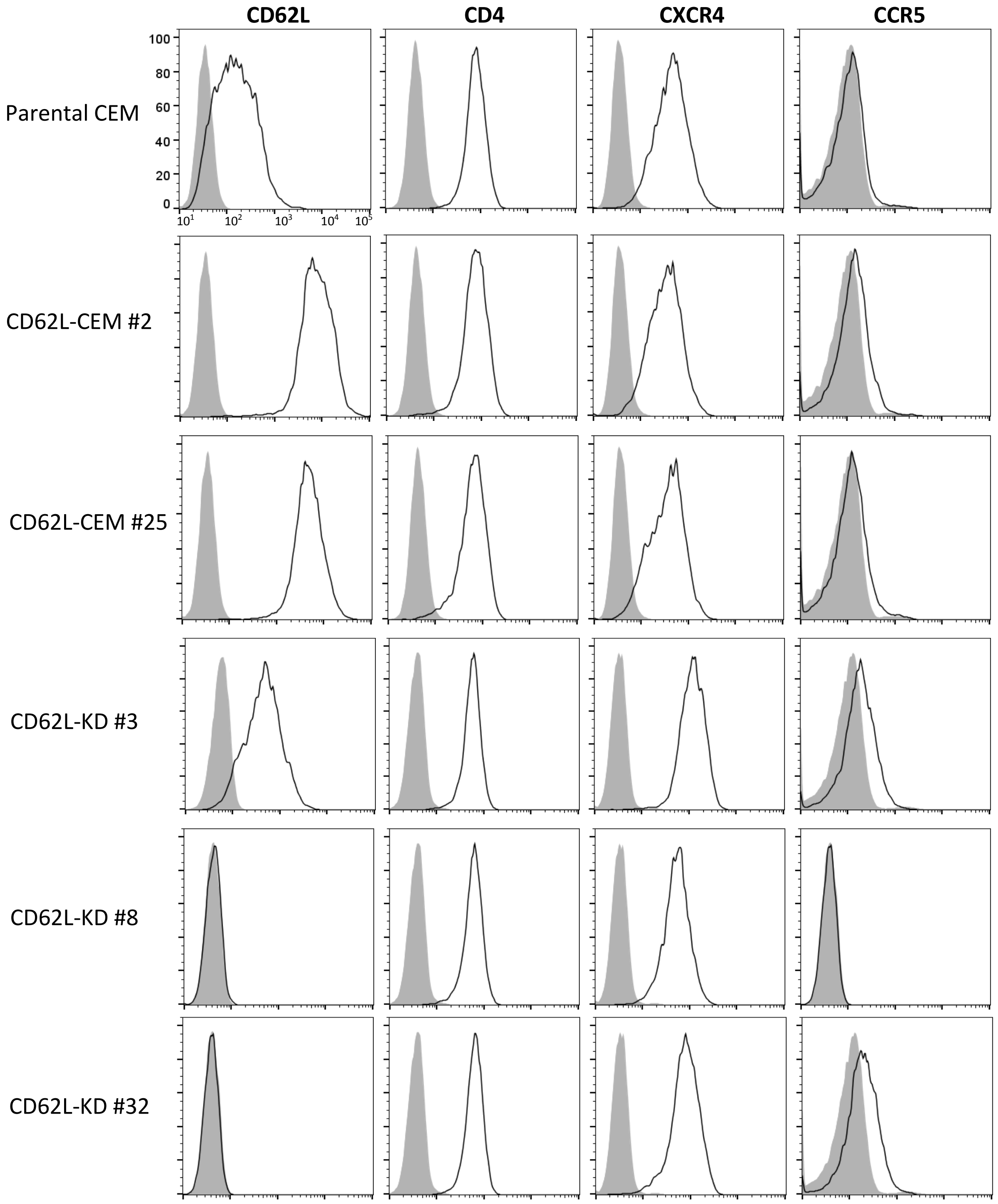

Supplementary Figure 4. Expression of CD62L, CD4, CXCR4 and CCR5 (solid line) and their isotype controls (shaded) in parental, CD62L-transfected and CD62L knockdown Rev-CEM cells. The results are representative of at least two independent experiments.

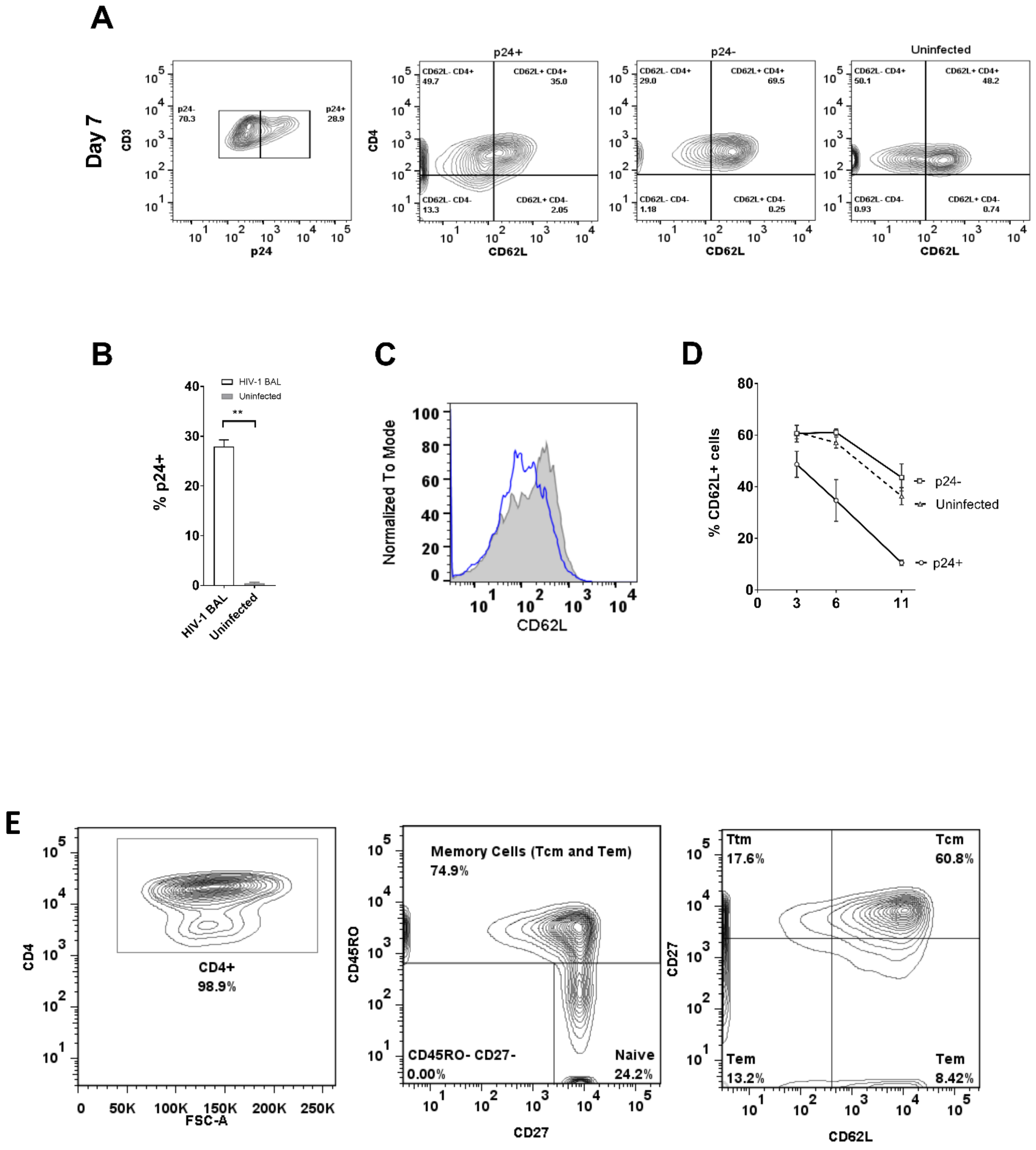

Supplementary Figure 5. HIV infection resulted in L-selectin shedding. A-C) Shedding of L-selectin (Fig 4E) results in reduced L-selectin expression on $p24^+$ but not $p24^-$ and uninfected cells on day 7 p.i. (A). The infection level was shown as % of $p24^+$ cells (B), and the corresponding L-selectin expression was shown in histograms for HIV-1_{BAL} infected (blue) and uninfected (gray) PBMC on day 7 p.i. (C). D) HIV-1_{BAL} infection resulted in the loss of L-selectin

expression as measured by % of $CD62L^+$ cells in $p24^+$ compared to $p24^-$ and uninfected PBMC on days 3, 6 and 11 p.i.   E) Memory T Cell Gating Scheme. Cells were gated $CD3^+$ (not shown), $CD4^+$ and then on $CD45RO^+$ and $CD27^{+/-}$ to isolate the memory cell population. Within the memory cell gate, samples were analyzed looking at CD62L vs CD27 to determine T_{CM} and T_{EM} populations, as well as the T_{TM} population described in the manuscript. Within the memory cell population we also examined CCR7 expression to further clarify memory cell subsets. The results are representative of at least two independent experiments.

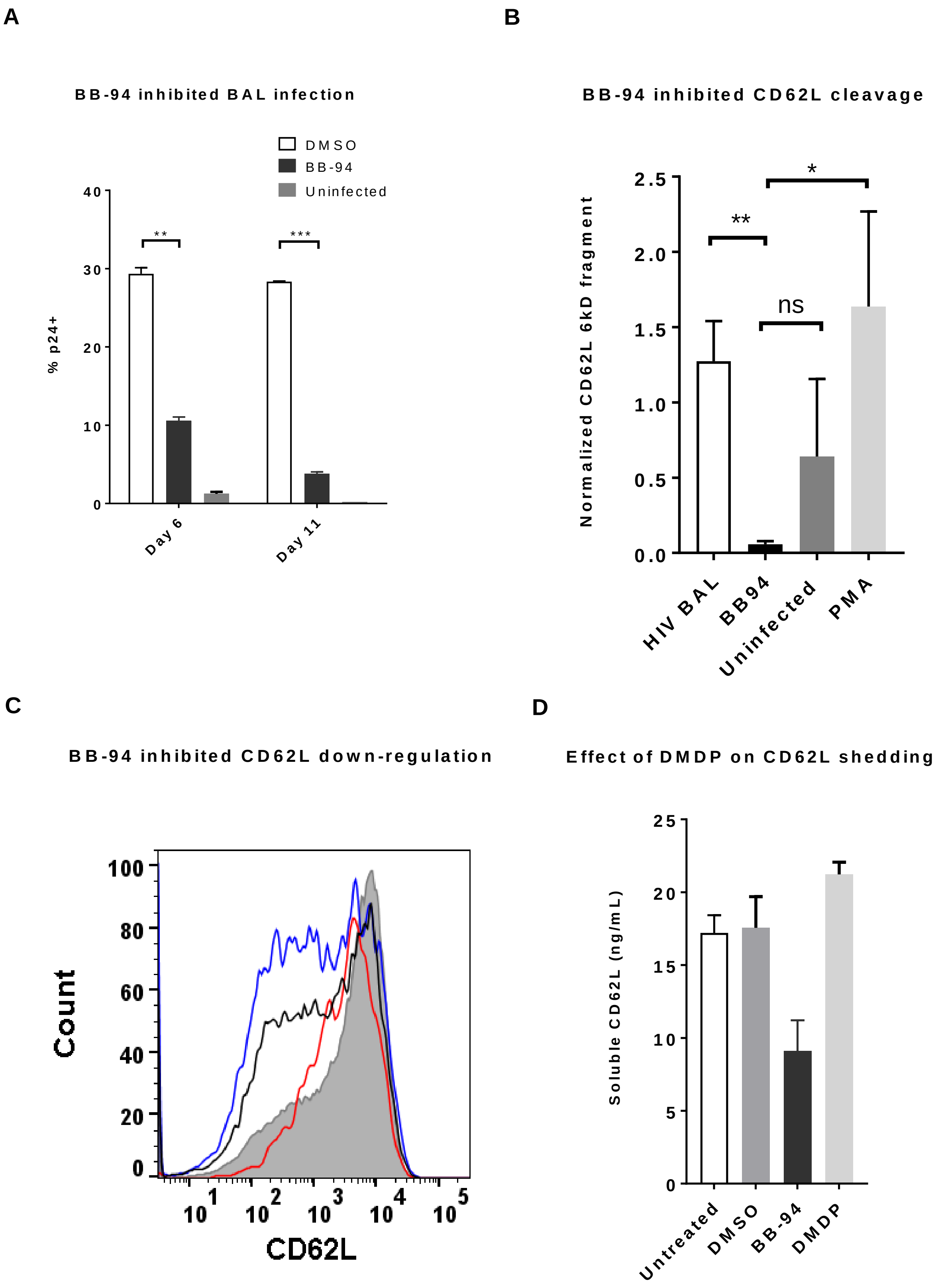
A
BB-94 inhibited BAL infection
DMSO
BB-94
Uninfected
% p24+
0
10
20
30
40
**
***
Day 6
Day 11
B
BB-94 inhibited CD62L cleavage
Normalized CD62L 6kD fragment
0.0
0.5
1.0
1.5
2.0
2.5
**
ns
*
HIV BAL
BB94
Uninfected
PMA
C
BB-94 inhibited CD62L down-regulation
Count
0
20
40
60
80
100
CD62L
10 1
10 2
10 3
10 4
10 5
D
Effect of DMDP on CD62L shedding
Soluble CD62L (ng/mL)
0
5
10
15
20
25
Untreated
DMSO
BB-94
DMDP

E

BB-94 inhibited CD62L shedding

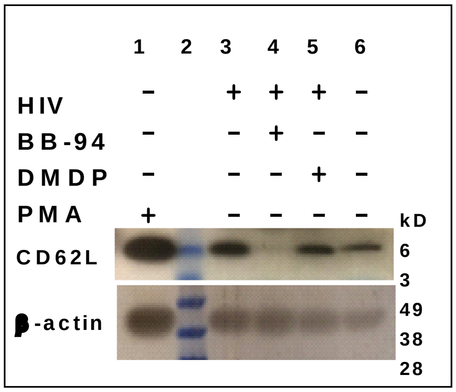

F

BB-94 inhibited HIVBAL infection

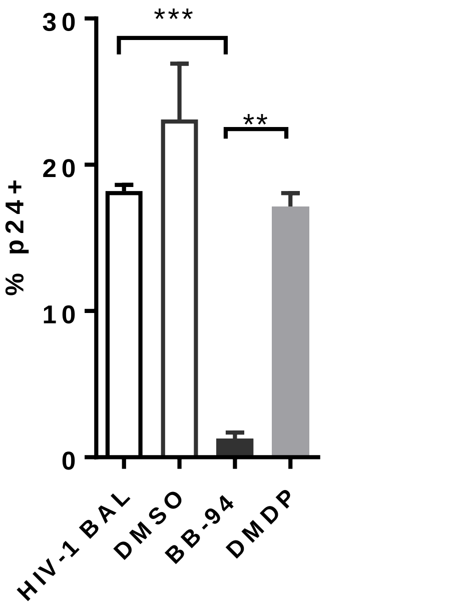

G

Effect of BB-94 on VSV infections

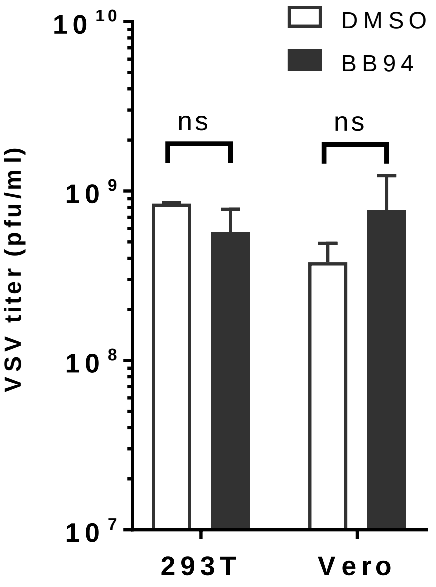

Supplementary Figure 6. Inhibition of L-selectin shedding and HIV infection. A) PBMC were infected with HIV-1_{BAL} with or without 5µM BB-94 and assessed for intracellular p24 expression on Day 6 and Day 11 p.i. DMSO was used as the vehicle control. B) Quantification of the cleaved 6kD L-selectin C-terminal fragment from three independent western blot experiments as those of Fig 6G. The intensity of the 6kD L-selectin band is presented as fold changes normalized against that of β-actin. C) L-selectin expression during HIV-1_{BAL} infection. The histograms correspond to the uninfected (gray area) and HIV-1_{BAL} infected PBMC in the absence (blue lines) or presence of 100uM BB-94 (red), or DMDP (black). D) The concentration of soluble CD62L present in cultured CD4 T cells treated with BB-94, DMDP and control DMSO. E) Western blot analysis showing BB-94 but not DMDP inhibited the selectin shedding. F) BB-94 but not DMDP inhibited HIV-1_{BAL} infections. G) 293T or Vero cells were infected with 1/5000 dilution of VSV stock virus in the presence of DMSO or BB94 for 48 hours. The titer of VSV in infected supernatants were determined using a plaque assay with 10^{-7} to 10^{-8} dilution of the supernatants. Panels A-F are from representative of two experiments.
